# Clinical Implications of Stroke Progressor Phenotypes defined based on ASPECTS decay and perfusion estimated infarct growth rate: insights from a large national thrombectomy registry

**DOI:** 10.1007/s10072-026-09148-4

**Published:** 2026-06-16

**Authors:** Antonio Ciacciarelli, Ettore Nicolini, Valentina Saia, Giovanni Pracucci, Umberto Pensato, Manuela De Michele, Lorenzo Benedetti, Ilaria Casetta, Enrico Fainardi, Simona Marcheselli, Valerio Da Ros, Ilaria Maestrini, Luigi Simonetti, Andrea Zini, Sergio Lucio Vinci, Paolino La Spina, Antonio Laiso, Patrizia Nencini, Bruno Del Sette, Tiziana Benzi Markushi, Sandra Bracco, Rossana Tassi, Andrea Saletti, Alessandro De Vito, Andrea Boghi, Andrea Naldi, Nicola Burdi, Giovanni Boero, Stefano Vallone, Guido Bigliardi, Guido Andrea Lazzarotti, Nicola Giannini, Nicola Milazzo, Alessandra Persico, Maria Ruggiero, Marco Longoni, Roberto Menozzi, Alessandro Pezzini, Luca Allegretti, Tiziana Tassinari, Francesca Mambrin, Manuel Cappellari, Mauro Bergui, Giovanni Bosco, Alessio Comai, Enrica Franchini, Emilio Lozupone, Marcella Caggiula, Sergio Zimatore, Marco Petruzzellis, Ivan Gallesio, Delfina Ferrandi, Marco Perri, Federica De Santis, Edoardo Puglielli, Alfonsina Casalena, Gianluca Galvano, Eleonora Saracco, Massimiliano Allegritti, Stefano Caproni, Matteo Alberti, Paolo Invernizzi, Giuseppe Carità, Monia Russo, Michele Besana, Alessia Giossi, Marco Filizzolo, Marina Mannino, Giuseppe Pelle, Michele Alessiani, Daniel Konda, Fabrizio Sallustio, Salvatore Mangiafico, Danilo Toni

**Affiliations:** 1 Emergency Department, Stroke Unit, Policlinico Umberto I, Sapienza University of Rome, Rome, Italy; 2 Department of Human Neuroscience, Sapienza University of Rome, Rome, Italy; 3 Neurology and Stroke Unit, S. Corona Hospital, Pietra Ligure, Italy; 4 Department of NEUROFARBA, Neuroscience Section, University of Florence, Florence, Italy; 5 Department of Neurology, IRCCS Humanitas Research Hospital, Rozzano, Milan, Italy; 6 Department of Biomedical Sciences, Humanitas University, Pieve Emanuele, Milan, Italy; 7 Neurology Unit, University Hospital Arcispedale S. Anna, Ferrara, Italy; 8 Dipartimento di Scienze Biomediche, Sperimentali e Cliniche, Università degli Studi di Firenze, Ospedale Universitario Careggi, Neuroradiologia, Florence, Italy; 9 Dipartimento di Biomedicina e Prevenzione, Università degli studi di Roma Tor Vergata, Rome, Italy; 10 UOSD Stroke Unit, Policlinico Tor Vergata, Rome, Italy; 11 IRCCS Istituto di Scienze Neurologiche di Bologna, Maggiore Hospital, Bologna, Italy; 12 UOC Neuroradiologia, Università di Messina, Messina, Italy; 13 UOSD Stroke Unit, Azienda Ospedaliero-Universitaria Messina, Messina, Italy; 14 SOD Interventistica Neurovascolare, AOU Careggi, Florence, Italy; 15 SOD Stroke Unit, Azienda Ospedaliero-Universitaria Careggi, Florence, Italy; 16 IRCCS Ospedale Policlinico San Martino, Genova, Italy; 17 UOC Neuroradiologia Diagnostica e Terapeutica Azienda Ospedaliero-Universitaria Senese, Siena, Italy; 18 UOC Stroke Unit, Azienda Ospedaliero-Universitaria Senese, Siena, Italy; 19 UO Neuroradiologia Dipartimento di Neuroscienze AZOU Ferrara, Ferrara, Italy; 20 UO Neurologia Provinciale SS Stroke Unit AOU Ferrara, Ferrara, Italy; 21 Neuroradiologia Ospedale S.G. Bosco, ASL città di Torino, Torino, Italy; 22 Neurology Unit, San Giovanni Bosco Hospital, Turin, Italy; 23 Neuroradiologia Ospedale SS. Annunziata ASL TA – Taranto, Taranto, Italy; 24 Neurologia-Stroke Unit Ospedale SS. Annunziata ASL TA – Taranto, Taranto, Italy; 25 Neuroradiologia, Ospedale Civile di Baggiovara, Azienda Ospedaliero-Universitaria di Modena, Modena, Italy; 26 Neurologia-Stroke Unit, Ospedale Civile di Baggiovara, Azienda Ospedaliero-Universitaria di Modena, Modena, Italy; 27 Neuroradiologia Azienda Ospedaliero Universitaria Pisana (AOUP), Pisa, Italy; 28 UOC Neurologia - Azienda Ospedaliero-Universitaria Pisana (AOUP)UOC Neurologia - Azienda Ospedaliero-Universitaria Pisana (AOUP), Pisa, Italy; 29 SS Interventistica Neurovascolare Fondazione IRCCS Policlinico San Matteo Pavia, Pavia, Italy; 30 UOC Neurologia d Urgenza e Stroke Unit, IRCCS C. Mondino – Pavia, Pavia, Italy; 31 Neuroradiologia Interventistica, AUSL Romagna Cesena, Cesena, Italy; 32 UOC Neurologia e Stroke Unit - Ospedale Bufalini Cesena- Azienda USL della Romagna, Cesena, Italy; 33 Dipartimento ad attività integrata interaziendale Diagnostico, Azienda Ospedaliero Universitaria, Parma , Italy; 34 Dipartimento di medicina e chirurgia, Università degli studi di Parma & percorso stroke care, dipartimento di emergenza-urgenza, azienda ospedaliero-universitaria, Parma, Italy; 35 SC Neuroradiologia Osp. Santa Corona Pietra Ligure Asl 2 Sistema Sanitario Regione Liguria, Pietra Ligure, Italy; 36 Neuroradiologia - Azienda Ospedaliera Universitaria Integrata Verona, Verona, Italy; 37 Stroke Unit - Azienda Ospedaliera Universitaria Integrata Verona, Verona, Italy; 38 Dip Neuroscienze Universitá di Torino, Torino, Italy; 39 Stroke Unit AOU Città della Salute - Ospedale Molinette – Torino, Torino, Italy; 40 Neuroradiologia, Ospedale Provinciale di Bolzano (SABES-ASDAA), Bolzano-Bozen, Bolzano, Italy; 41 Neurologia-Stroke Unit, Ospedale Provinciale di Bolzano (SABES-ASDAA), Bolzano-Bozen, Bolzano, Italy; 42 Neuroradiologia Ospedale Vito Fazzi SL LE – Lecce, Lecce, Italy; 43 U.O.C Neurologia/ U.O.S. Stroke Unit ospedale Vito Fazzi asl Lecce, Lecce, Italy; 44 Neuroradiologia AOU Consorziale Policlinico Bari, Bari, Italy; 45 UOC Neurologia Universitaria e Stroke Unit F. Puca AOU Consorziale Policlinico, Bari, Italy; 46 Radiologia e Interventistica AOU Alessandria, Alessandria, Italy; 47 Neurologia AOU Alessandria, Alessandria, Italy; 48 UOC Radiologia diagnostica e interventistica, p.o. SS Filippo e Nicola Avezzano, Avezzano, Italy; 49 UOC NEUROLOGIA E STROKE UNIT p.o. SS. Filippo e Nicola Avezzano, Avezzano, Italy; 50 ASL TERAMO, Teramo, Italy; 51 UOC Diagnostica per Immagini, Radiologia Interventistica e Neuroradiologia, ARNAS Garibaldi, Catania, Italy; 52 UOC Neurologia, Arnas Garibaldi Catania, Catania, Italy; 53 Dipartimento di Diagnostica, Azienda Ospedaliera S. Maria, Radiologia Interventistica, Terni, Italy; 54 Dipartimento di Neuroscienze, Azienda Ospedaliera S. Maria, Neurologia e Stroke Unit, Terni, Italy; 55 Neuroradiologia Fondazione Poliambulanza di Brescia, Brescia, Italy; 56 Neurologia e stroke unit fondazione Poliambulanza di Brescia, Brescia, Italy; 57 UOC Neuroradiologia, Osp.Santa Maria Misericordia, Rovigo, Italy; 58 Stroke Unit ULSS5 Rovigo, Rovigo, Italy; 59 S.C Neuroradiologia, Dipartimento di Neuroscienze, presidio ospedaliero di Cremona, Cremona, Italy; 60 UOC Neurologia ASST Cremona, Cremona, Italy; 61 UO Radiologia, Azienda Ospedaliera Ospedali Riuniti Villa Sofia-Cervello, Palermo, Italy; 62 UOC Neurologia, Azienda Ospedaliera Ospedali Riuniti Villa Sofia-Cervello, Palermo, Italy; 63 Neuroradiologia Interventistica, Ospedale SM Goretti, Latina, Italy; 64 UOC Neurologia, Ospedale SM Goretti, Latina, Italy; 65 UOC Radiologia Diagnostica e Interventistica Ospedale dei Castelli Asl Roma 6, Ariccia, Italy; 66 Unità Di Trattamento Neurovascolare, Ospedale Dei Castelli-ASLRM6, Rome, Italy; 67 IRCCS Neuromed, Pozzilli (IS), Pozzilli, Italy

**Keywords:** Infarct Growth Rate, ASPECTS, Fast Progressors, CT Perfusion Imaging, Stroke Phenotypes

## Abstract

**Introduction:**

Infarct growth rate (IGR) is highly heterogeneous among ischemic stroke patients, reflecting a spectrum of progressor phenotypes with clinical implications. We aim to compare different imaging approaches to investigate stroke progressors phenotypes and their clinical implications in patients undergoing thrombectomy.

**Methods:**

Data are from the prospective Italian Registry of Endovascular Treatment in Acute Stroke (IRETAS). Patients with M1/M2 occlusion and known symptom onset were included. Progressor phenotypes were defined using (1) NCCT-based definitions (ASPECTS points decay per hour < 0.25 pts/h=slow progressor, 0.25–0.50 pts/h=intermediate, and > 0.50 pts/h=fast); and (2) CTP-based definitions (CTP-estimated core divided by time of onset < 5 mL/h=slow progressors, 5–10mL/h=intermediate, and > 10 mL/h=fast). The primary outcome was 90-day good functional outcome (modified Rankin Scale [mRS] = 0–2). Associations were assessed with logistic regression analyses adjusted for age, sex, NIHSS, TICI score, thrombolysis, and imaging-to-recanalization time.

**Results:**

Of 26799 patients screened, 8322 (31.1%) were included (NCCT group: 8076; CTP group: 897 patients). NCCT-based progressor phenotype was associated with lower odds of good outcome (aOR 0.82 [95%CI = 0.72–0.92] per each progressor phenotype increase). ASPECTS decay per hour was associated with lower odds of good outcome (acOR 0.94 [95%CI = 0.89–0.99]). No significant association was observed for either CTP-based progressor phenotype or CTP-based IGR (mL/h). Similar findings were observed for secondary outcomes.

**Conclusions:**

In this large, real-world cohort of stroke patients, NCCT-based IGR was associated with functional outcomes, whereas CTP-based IGR was not. This highlights the need to refine and identify more accurate markers of infarct growth within perfusion imaging.

**Supplementary Information:**

The online version contains supplementary material available at 10.1007/s10072-026-09148-4.

## Introduction

The infarct growth rate (IGR)—the speed at which the ischemic core expands over time—is highly heterogeneous among ischemic stroke patients, reflecting a spectrum of stroke progressor phenotypes [1–3]. Patients are often classified as fast progressors, who develop large infarcts early, and slow progressors, who maintain small infarct volumes even at delayed imaging [1]. While collateral circulation is a key determinant of IGR, additional factors, such as blood pressure, head position, temperature, blood glucose, genetic background, and comorbidities may influence the perfusion status and cerebral ischemic tolerance, i.e., the resilience of the cerebral tissue to hypoperfusion [4–11].

Endovascular thrombectomy (EVT) and intravenous thrombolysis represent the cornerstone of treatment for large vessel occlusion (LVO) stroke patients presenting within six hours from symptoms onset [12, 13]. Although IGR does not currently guide acute treatment decisions, it is a powerful prognostic marker, with fast progressors usually experiencing worse functional outcomes compared to slow progressors [3, 14, 15]. Growing interest in neuroprotective interventions [16] and adjunctive therapies to enhance cerebral ischemic tolerance and slow down IGR underscores the need for better characterization of progressor phenotypes, both to improve prognosis and patient selection for neuroprotection strategies [17, 18]. Different imaging methods have been proposed to assess the IGR, with the most common approaches involving direct visualization of parenchymal ischemic changes (e.g., Computed Tomography [CT] hypodensity) or by estimation of ischemic core volume using perfusion imaging techniques, divided by the time of symptom onset [14, 16]. Recently, there has been increasing interest in a more granular characterization of stroke progressor phenotypes, including the identification of intermediate categories [14]. However, direct comparisons among imaging techniques and studies assessing these more detailed phenotypes remain limited [1, 3].

We aim to compare different imaging approaches to investigate stroke progressors phenotypes and their clinical implications in a large Italian population of ischemic stroke patients treated with EVT.

## Methods

### Patient sample and study design

Patients are from the IRETAS registry, which is a national, multicentre, prospective study of patients treated with EVT across 45 Italian centers. Details on the study design and inclusion criteria have been published elsewhere [19]. Data from January 2011 to December 2023 were analyzed. For this study, we included adult patients with (i) occlusion of the M1 and/or M2 segment of the middle cerebral artery (MCA), (ii) known time of stroke symptom onset, (iii) pre-morbidity modified Rankin Scale (mRS) ≤ 2, and (iv) neuroimaging available at baseline to calculate at least one between perfusion-estimated ischemic core on CT perfusion (CTP) or Alberta Stroke Program Early CT Score (ASPECTS) on non-contrast CT (NCCT). We limited our inclusion criteria to occlusions of the M1 and/or M2 segments of the MCA to achieve a homogeneous study population concerning the affected vascular territory and ASPECTS. Neurologists and neuroradiologists, respectively, collected clinical and radiological data. According to current international guidelines, the stroke team (neuroradiologist and neurologist) of individual centers decided on the EVT eligibility and technique [20].

### Imaging acquisition and analysis

CT angiography (CTA) or Magnetic Resonance Angiography (MRA) assessed the site of vessel occlusions on admission. Hemorrhagic transformation was evaluated at 24 h on CT or MRI scans according to European Cooperative Acute Stroke Study (ECASS) II criteria [21]. Neuroradiologists assessed reperfusion status at the end of the EVT procedure according to the thrombolysis in cerebral infarction (TICI) score [22]. Successful recanalization was defined as TICI≥2b. CTP imaging was used to identify potentially salvageable brain tissue using fully automated software, chosen at each center’s discretion (Table S1). Ischemic core was defined as the area with relative cerebral blood flow (rCBF) < 30% of normal tissue. Critically hypoperfused tissue was defined as region in which time to maximum (Tmax) > 6 s. The difference between Tmax > 6 s and rCBF < 30% (mismatch volume) was considered penumbra, and their ratio defined as mismatch ratio.

### Stroke progressor phenotypes definitions

Based on a recently proposed definition of stroke progressors [3], we classified patients into fast (IGR > 10mL/h), intermediate (IGR = 5-10mL/h), and slow progressors (IGR < 5mL/h). To estimate IGR, we adopted two different methods that leverage indirect (severe hypoperfusion on CTP) and direct (hypodensity on NCCT) visualization of the ischemic core:

1. CTP-based stroke progressor phenotypes: CTP-estimated IGR was calculated by dividing the baseline CTP-estimated ischemic core volume by onset-to-imaging time (mL/h). Patients were categorized as fast progressors (IGR ≥ 10 mL/h), intermediate progressors (IGR between 5 and 10 mL/h), and slow progressors (IGR < 5 mL/h) [3].
2. NCCT-based stroke progressor phenotypes [16, 23]: NCCT-estimated IGR was estimated by subtracting the baseline ASPECTS from 10 and dividing by onset-to-imaging time (ASPECTS points decay per hour). Assuming the MCA territory encompasses approximately 200 mL of brain tissue [24], each ASPECTS point was estimated to represent 20 mL. Based on the rate of ASPECTS decay per hour, patients were categorized as fast progressors (> 0.5 pts/h), intermediate progressors (0.25–0.5 pts/h), and slow progressors (< 0.25 pts/h)—corresponding approximately to > 10mL/h, 5-10mL/h, and < 5mL/h of IGR, respectively.

### Outcome

The primary outcome was a good functional outcome at 90 days (mRS of 0–2). The secondary outcomes included moderate functional outcome at 90 days (mRS of 0–3), the ordinal mRS at 90 days, mortality at 90 days, any intracerebral hemorrhage (ICH), and symptomatic intracerebral hemorrhage (sICH).

### Statistical analysis

Continuous variables were expressed as medians and interquartile ranges (IQRs), whereas the categorical variables were reported as counts and percentages. Baseline characteristics and outcomes were described and compared across stroke progressor phenotypes using the Kruskal-Wallis, Pearson χ2 test, or Fisher’s exact test as appropriate.

Associations between stroke progressor phenotypes and dichotomized outcomes (mRS, any ICH, sICH) were assessed with binary logistic regression, whereas associations with ordinal 90-day mRS were assessed with ordinal logistic regression. Both unadjusted analyses and adjusted analyses were performed. All adjusted analyses included age, sex, baseline National Institutes of Health Stroke Scale (NIHSS), recanalization status (TICI score), imaging-to-recanalization time, and intravenous thrombolysis bridging as adjustment variables. Effect size estimates derived from logistic regression analyses were reported as odds ratio (OR) with 95% confidence intervals (95%CI). The OR reported in this study represent the odds ratios of a worse functional outcome (ordinal mRS) or achieving an outcome (e.g., mRS 0–2 at 90 days) associated with each stepwise increase in stroke progressor phenotype (i.e., from slow to intermediate or from intermediate to fast progressor). Stroke progressor phenotype was treated as an ordinal independent variable, assuming a stepwise effect across categories. The assumption of proportional odds was verified with the Brant test for the ordinal logistic regression analyses.

Since categorization of the study cohort in three categories may result in information loss, we performed sensitivity analyses in which associations between the primary outcome and IGR were evaluated using ASPECTS decay per hour and CTP-estimated IGR as continuous variables. OR were reported per each ASPECTS point decay per hour increase and per each 5-mL CTP-estimated IGR increase, respectively. Logistic regression analyses were otherwise identical to the main analyses.

No imputation was performed based on the assumption that data were not missing at random. All calculated p-values were two-tailed and statistical significance was assumed at *p* < 0.05. The statistical analysis was performed with SPSS (Version 29.0).

## Results

### Patient characteristics

**Fig. 1:**
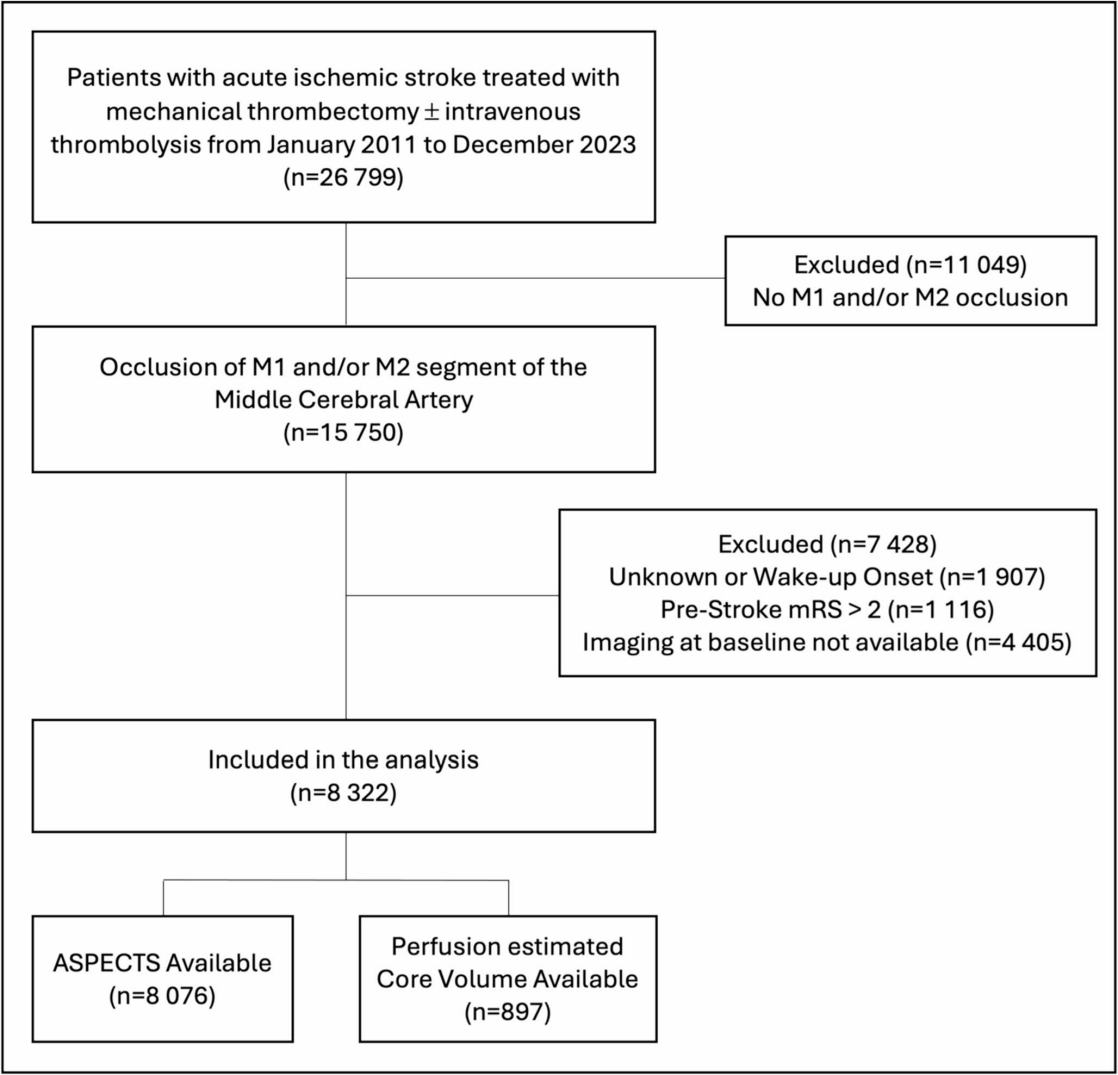
Flowchart of included patients

**Fig. 2:**
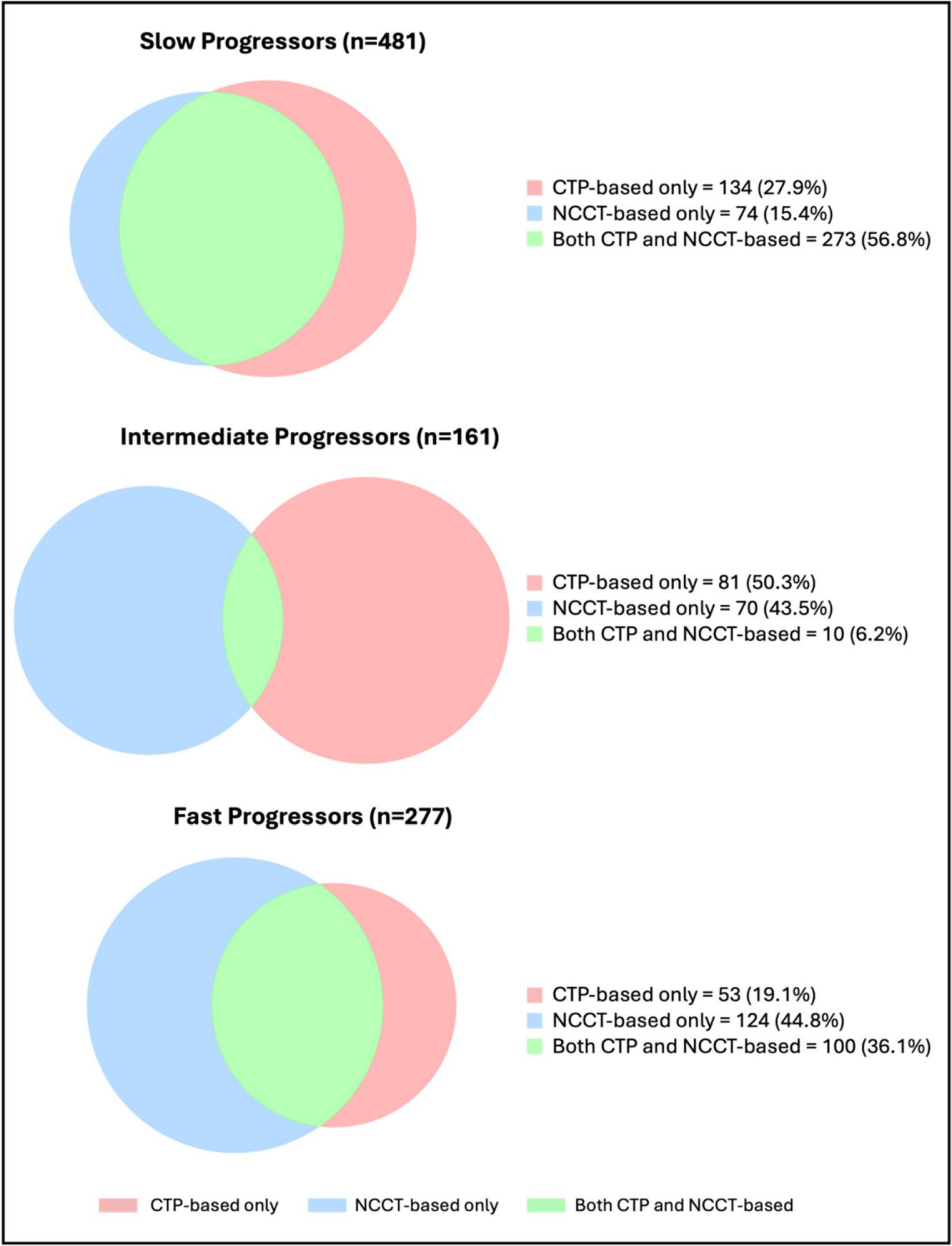
Venn diagrams showing the overlap between NCCT-based and CTP-based classification of slow, intermediate, and fast progressors among patients with both imaging modalities available. For each progressor category, the left (blue) area represents patients classified according to NCCT only, the right (red) area represents patients classified according to CTP only, and the overlapping (green) area represents patients classified in the same category by both methods. Percentages are calculated within each progressor category

Of the26799 patients enrolled in the IRETAS study, 11040 (41.2%) were excluded due to vessel occlusion outside the M1 or M2 segments, 1907 (7.1%) were excluded due to unknown time of symptom onset, 1116 (4.2%) due to a baseline mRS > 2, and 4405 (16.4%) due to unavailable baseline imaging. Overall, 8322 (31.1%) patients met the inclusion criteria and were included in this analysis. Among these, 897 (10.8%) had baseline CTP available and were included in the analysis for the CTP-based definition, while 8076 (97.0%) patients had baseline ASPECTS available and were included in the analysis for the NCCT-based definition (Fig. 1). In the CTP-based IGR group, the median age was 76 years (IQR 68.5–84.7), 57.5% were female, and the median IGR was 8.1 (IQR 0.5–15.4). In the NCCT-based group, the median age was 77 years (IQR 67.2–83.2), 55.9% were female, and the median ASPECTS decay was 0.0 (IQR 0.0–0.65). Using the CTP-based definition, 570 (63.5%) were classified as slow progressors, 111 (12.4%) as intermediate, and 216 (24.1%) as fast progressors. Using NCCT-based definition, 4713 (58.4%) were classified as slow progressors, 900 (11.1%) as intermediate progressors, and 2463 (30.5%) as fast progressors. Figure 2 illustrates the overlap between NCCT-based and CTP-based classifications of slow, intermediate, and fast progressors among patients with both assessments available. Concordance was highest for slow progressors, with 56.8% classified as slow by both definitions. Agreement was lower for fast progressors (36.1%) and minimal for intermediate progressors (6.2%).

### Comparison of baseline characteristics across stroke progressor phenotypes

Baseline demographics, medical history, clinical features, treatment and neuroimaging characteristics are shown in Table 1. Fast progressors were younger than intermediate and slow progressors in both definitions [NCCT: 76 years (IQR 65.6–82.6) vs. 77 years (IQR 67.9–83.2) vs. 77 years (IQR 67.8–83.4), *p* < 0.001; CTP: 74 years (IQR 63.5–82.6) vs. 79 years (IQR 70.3–85.1) vs. 79 years (IQR 70.1–85.1), *p* < 0.001], and had higher baseline NIHSS [NCCT: 17 (IQR 13–20) vs. 15 (IQR 10–19) vs. 14 (IQR 9–18), *p* < 0.001; CTP: 17 (IQR 13–21) vs. 15 (IQR 10–20), vs. 11 (IQR 7–17), *p* < 0.001]. CTP-estimated core volume, total hypoperfusion volume, ASPECTS, and onset-to-imaging time were different across groups, with fast progressors consistently exhibiting more extensive signs of ischemic damage at baseline (i.e., larger CTP-estimated core volumes, larger total hypoperfusion volume, and lower ASPECTS) and shorter symptom onset-to-imaging time (Table 1). Missing data are shown in Table S2.

**Table 1:** Comparison of Baseline Clinical Features Among Slow, Intermediate, and Fast Progressors Across Modalities

	ASPECTS dcay per hour				CTP-based IGR			
	Slow (n=4713)	Intermediate (n=900)	Fast (n=2463)	P value	Slow (n=570)	Intermediate (n=111)	Fast (n=216)	P value
Demographics and Medical History								
Age, y (median, IQR)	77 (67.8-83.4)	77 (67.9-83.2)	76 (65.6-82.6)	**<0.001**	79 (70.1-85.1)	79 (70.3-85-1)	74 (63.5-82.6)	** <0.001**
Sex (female), n (%)	2635 (55.9%)	533 (59.2%)	1346 (54.6%)	0.061	348 (61.1%)	60 (54.1%)	107 (49.5%)	** 0.011**
Previous TIA/Stroke, n (%)	176 (3.7%)	20 (2.2%)	89 (3.6%)	0.076	16 (2.8%)	3 (2.7%)	6 (2.8%)	1.000
Atrial Fibrillation, n (%)	1684 (35.7%)	334 (37.1%)	847 (34.4%)	0.293	208 (36.5%)	46 (41.4%)	79 (36.6%)	0.609
Diabetes, n (%)	716 (15.2%)	152 (16.9%)	392 (15.9%)	0.383	93 (16.3%)	18 (16.2%)	35 (16.2%)	1.000
Hypertension, n (%)	3057 (64.9%)	586 (65.1%)	1611 (65.4%)	0.899	398 (69.8%)	79 (71.2%)	157 (72.7%)	0.734
Coronary Artery Disease, n (%)	469 (10.0%)	90 (10.0%)	289 (11.7%)	0.057	80 (14.0%)	11 (9.9%)	29 (13.4%)	0.526
Valvulopathy, n (%)	301 (6.4%)	56 (6.2%)	158 (6.4%)	0.979	34 (6.0%)	5 (4.5%)	7 (3.2%)	0.296
Heart Failure, n (%)	302 (6.4%)	60 (6.7%)	187 (7.6%)	0.164	30 (5.3%)	10 (9.0%)	14 (6.5%)	0.320
Smokers (current or past), n (%)	657 (13.9%)	129 (14.3%)	393 (16.0%)	0.070	50 (8.8%)	13 (11.7%)	37 (17.1%)	** 0.004**
Hyperlipemia, n (%)	1171 (24.8%)	255 (28.3%)	677 (27.5%)	** 0.013**	173 (30.4%)	39 (35.1%)	87 (40.3%)	** 0.027**
Carotid Atherosclerosis, n (%)	96 (2.0%)	25 (2.8%)	83 (3.4%)	** 0.003**	28 (4.9%)	8 (7.2%)	9 (4.2%)	0.488
Cancer, n (%)	302 (6.4%)	59 (6.6%)	162 (6.6%)	0.957	47 (8.2%)	7 (6.3%)	13 (6.0%)	0.515
Antiplatelet therapy, n (%)	1255 (26.6%)	245 (27.2%)	706 (28.7%)	0.184	170 (29.8%)	26 (23.4%)	63 (29.2%)	0.391
Anticoagulation therapy, n (%)	803 (17.0%)	161 (17.9%)	451 (18.3%)	0.883	113 (19.8%)	19 (17.1%)	35 (16.2%)	0.847
Antihypertensive therapy, n (%)	2506 (52.2%)	487 (54.1%)	1323 (53.7%)	0.830	349 (61.2%)	56 (50.5%)	124 (57.4%)	0.094
Statins, n (%)	820 (17.4%)	179 (19.9%)	434 (17.6%)	0.197	131 (23.0%)	23 (20.7%)	53 (24.5%)	0.743
Clinical, Treatment, and Neuroimaging Characteristics								
Baseline NIHSS (median, IQR)	14 (9-18)	15 (10-19)	17 (13-20)	**<0.001**	11 (7-17)	15 (10-20)	17 (13-21)	**<0.001**
Intravenous thrombolysis, n (%)	2374 (52.2%)	398 (45.1%)	1295 (54.0%)	**<0.001**	264 (47.8%)	67 (60.4%)	122 (57.0%)	**0.010**
TICI 2b-3, n (%)	3795 (82.3%)	708 (79.6%)	1885 (77.8%)	**<0.001**	488 (85.8%)	85 (78.0%)	177 (83.5%)	0.113
Onset-to-Imaging, min (median, IQR)	136 (90-248)	212 (151-322)	118 (88-182)	**<0.001**	217 (118-473)	141 (97-219)	96 (72-132)	**<0.001**
Onset-to-Recanalization, min (median, IQR)	297 (227-398)	253 (286-460)	275 (220-341)	**<0.001**	348 (242-536)	301 (231-390)	255 (204-320)	**<0.001**
ASPECTS (median, IQR)	10 (10-10)	9 (8-9)	8 (7-8)	**<0.001**	9 (8-10)	8 (7-10)	9 (7-10)	**<0.001**
ASPECTS decay per hour (median, IQR)	0.0 (0.0-0.0)	0.38 (0.32-0.44)	1.0 (0.71-1.51)	**<0.001**	0.04 (0.0-0.38)	0.58 (0.0-1.05)	0.81 (0.0-1.38)	**<0.001**
CTP-estimated Core Volume, mL (median, IQR)	2.0 (0.0-15.0)	8.0 (1.5-20.5)	18.5 (5.0-37.0)	**<0.001**	0.0 (0.0-5.0)	16.7 (10-27.0)	35 (23.3-56.5)	**<0.001**
Hypoperfusion Volume, mL (median, IQR)	77 (47-125)	88 (51-122)	112 (70-153)	**<0.001**	74 (45-106)	109 (70-135)	133 (89-173)	**<0.001**
CTP-based IGR, mL/h (median, IQR)	0.9 (0.3-4.3)	2.0 (0.7-4.7)	112 (70-153)	**<0.001**	0.7 (0.28-1.78)	7.6 (5.88-8.57)	21.1 (14.1-30.4)	**<0.001**

Significant p-value are reported in bold. *TIA* Transitory Ischemic Attack.  *NIHSS* National Institutes of Health Stroke Scale. *TICI* Thrombolysis in Cerebral Infarction scale. A*SPECTS* Alberta Stroke Program Early CT Score. *IGR* Infarct Growth Rate

### Associations between stroke progressors phenotypes and outcomes

**Fig. 3:**
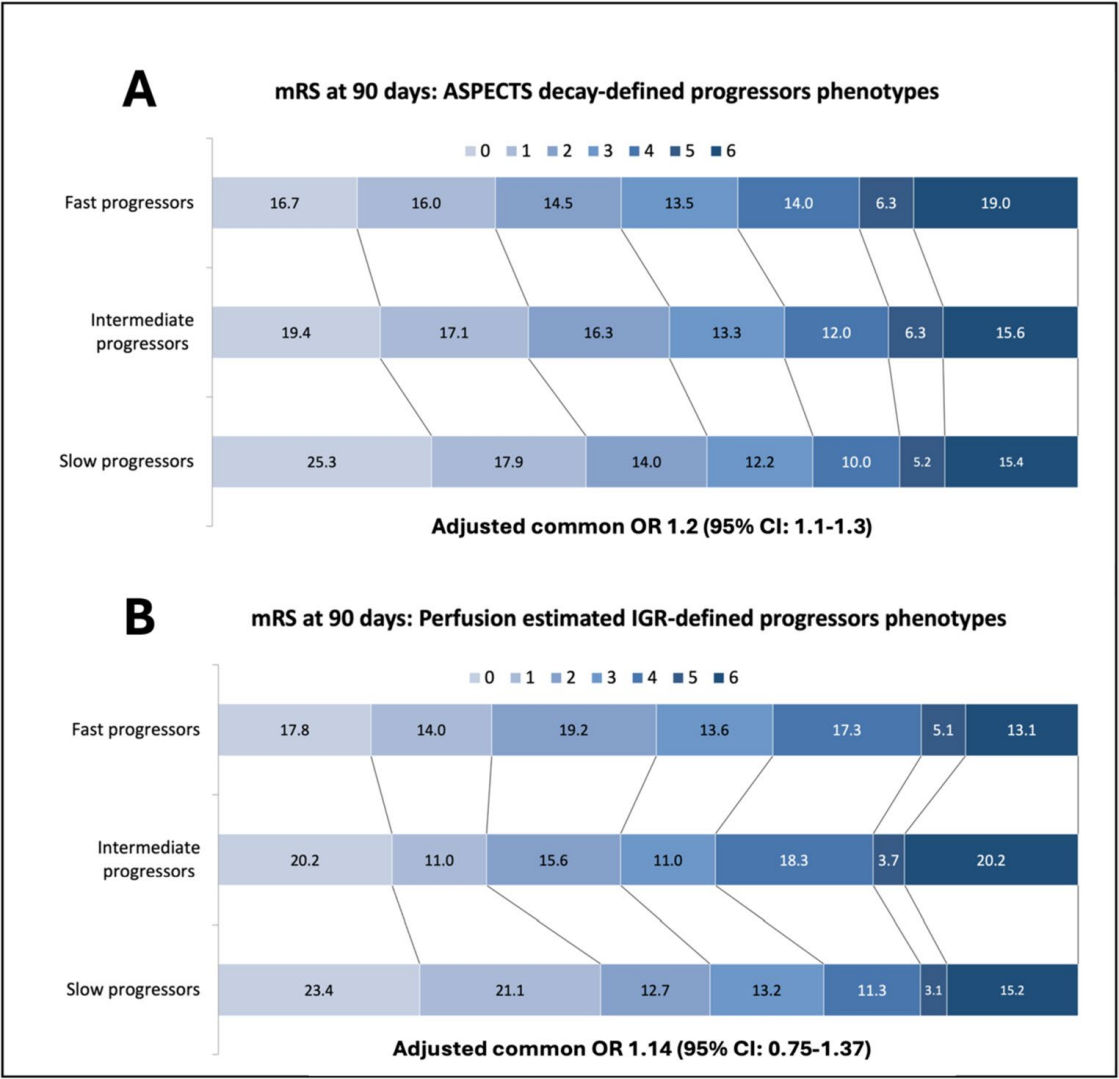
Ordinal mRS distribution stratified by stroke progressor phenotypes. Horizontal stacked bar graphs (Grotta Bars) show the distribution of modified Rankin Scale (mRS) score at 90 days by fast vs. intermediate vs. slow progressors according to ASPECTS decay (**A**), and Infarct Growth Rate (**B**) definitions. Bars are labeled with proportions

In the NCCT-based definition group, there was a significant association between stroke progressors phenotypes and mRS 0–2 (adjusted OR 0.82 [95% CI: 0.72–0.92] per each stroke progressor phenotype increase), as well as across all secondary outcomes (Table 2), with lower odds of achieving good outcomes consistently observed for increase progressor phenotype. In the CTP-based definition group, there was no association between stroke progressors phenotypes and mRS 0–2 (adjusted OR 0.85 [95% CI: 0.53–1.34]), as well as across all secondary outcomes (Table 2). There was a significant association between progressors phenotypes defined by NCCT-based definition and ordinal mRS at 90 days (adjusted common OR 1.2 [95% CI: 1.1–1.3]) whereas no significant association was observed between progressors phenotypes defined by CTP-based definition and ordinal mRS at 90 days (adjusted common OR 1.14 [95% CI: 0.75–1.37]) **(**Fig. 3**).**

**Table 2:** Comparison of outcomes among slow, intermediate, and fast progressors across modalities

Functional Outcomes	ASPECTS decay per hour				CTP-based Infarct Growth Rate			
	Slow (n = 4713)	Intermediate (n = 900)	Fast (n = 2463)	aOR (95% CI) [OR (95% CI)]	Slow (n = 570)	Intermediate (n> = 111)	Fast (n = 216)	aOR (95% CI) [OR (95% CI)]
mRS 0–2 at 90 days	2539 (57.2%)	444 (52.8%)	1093 (47.2%)	0.82 (0.72–0.92) [0.67 (0.61–0.74)]	315 (57.2%)	51 (46.8%)	109 (50.9%)	0.89 (0.52–1.52) [0.85 (0.53–1.34)]
mRS 0–3 at 90 days	3082 (69.4%)	556 (66.1%)	1405 (60.7%)	0.84 (0.74–0.95) [0.68 (0.61–0.76)]	388 (70.4%)	63 (57.8%)	138 (64.5%)	0.74 (0.42–1.31) [0.75 (0.47–1.21)]
Death at 90 days	682 (15.4%)	131 (15.6%)	439 (19.0%)	1.1 (0.94–1.27) [1.3 (1.1–1.5)]	84 (15.2%)	22 (20.2%)	28 (13.1%)	1.57 (0.79–3.14) [1.68 (0.91–3.10)]
mRS at 90 days*	2 (0–4)	2 (1–4)	3 (1–5)	1.2 (1.1–1.3) [1.5 (1.4–1.6)]	2 (1–4)	3 (1–4)	2 (1–4)	1.14 (0.75–1.37) [1.33 (1.02–1.72)]
Any ICH	1051 (23.1%)	248 (28.2%)	761 (31.8%)	1.36 (1.21–1.53) [1.6 (1.4–1.7)]	139 (24.9%)	38 (34.9%)	61 (28.6%)	1.60 (0.95–2.70) [1.33 (0.81–2.18)]
sICH	239 (5.2%)	52 (5.9%)	224 (9.4%)	1.61 (1.31–1.97) [1.9 (1.5–2.3)]	29 (5.2%)	11 (10.1%)	10 (4.7%)	2.38 (0.47–2.35) [2.28 (0.94–5.55)]

Adjusted for age, sex, baseline NIHSS, recanalization status, imaging-to-recanalization time, i.v. thrombolysis. aOR adjusted odds. OR unadjusted odds ratio. NIHSS National Institutes of Health Stroke Scale. ICeH Intracerebral Hemorrhage. sICH Symptomatic ICH. The OR reported represent the odds ratios of achieving an outcome associated with each stepwise increase in stroke progressor phenotype (i.e., from slow to intermediate or from intermediate to fast progressor). Numbers are n (%) for categorial variables and median (IQR) for continuous variables. * The mRS ordinal shift analyses are reported as adjusted and unadjusted common OR

### ﻿Sensitivity analysis

**Fig. 4:**
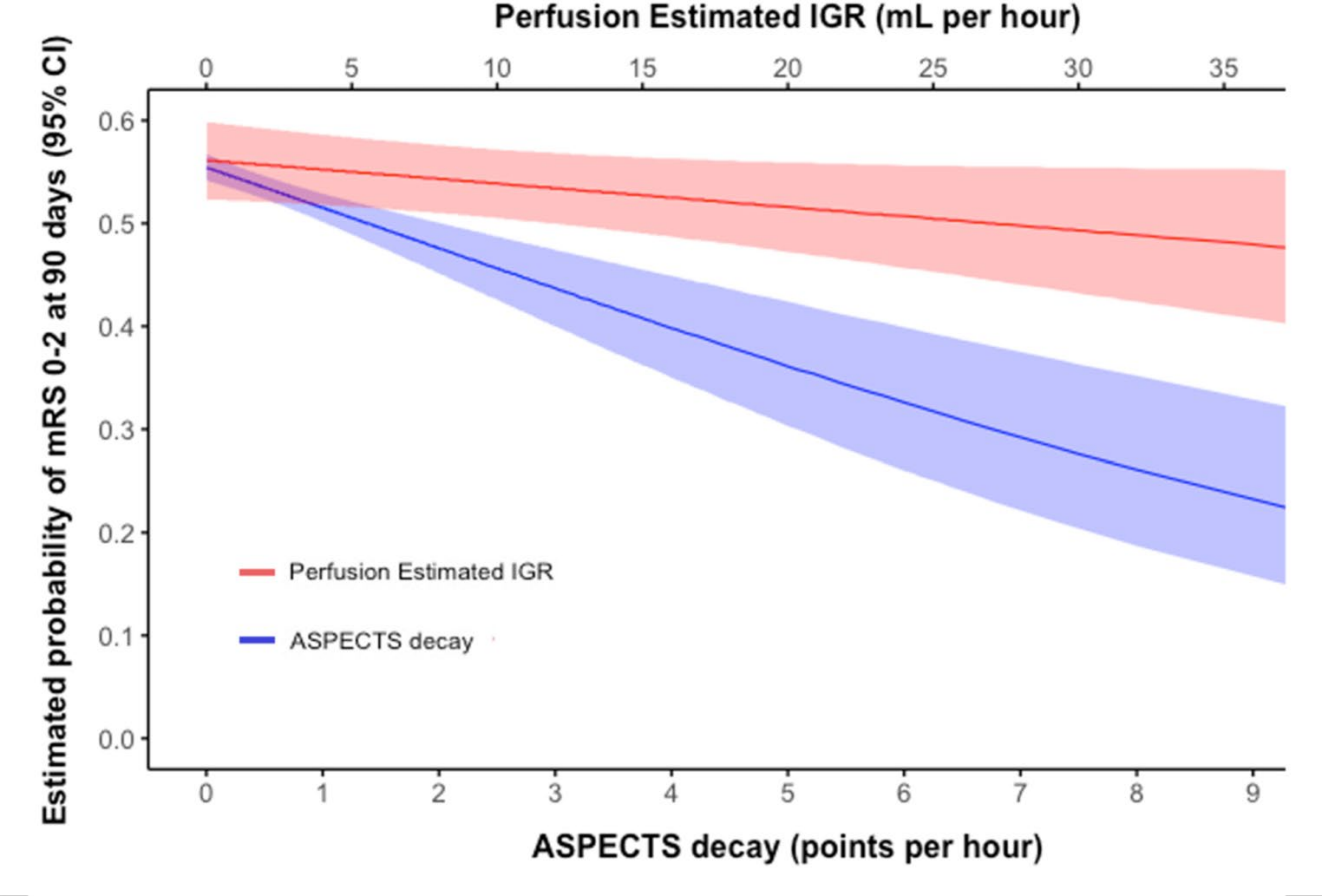
Association of Perfusion estimated IGR and ASPECTS decay and the estimated probability of mRS 0–2 at 90 days and 95% CI

There was a significant association between ASPECTS decay per hour and mRS 0–2 at 90 days (adjusted OR 0.94 [95% CI: 0.89–0.99] per 1-point ASPECTS decay per hour) and ordinal mRS at 90 days (adjusted common OR 1.03 [95% CI: 1.00-1.07] per 1-point ASPECTS decay per hour) **(**Fig. 4**)**.

There was no association between CTP-estimated IGR and mRS 0–2 at 90 days (adjusted OR 0.98 [95% CI: 0.92–1.03] per 5 mL/h CTP-estimated IGR increase) or ordinal mRS at 90 days (adjusted common OR 1.02 [95% CI: 0.98–1.06] per 5 mL/h CTP-estimated IGR increase) **(**Fig. 4**)**.

## Discussion

In this large, multicenter cohort of patients with MCA occlusion treated with EVT, we investigated stroke progressor phenotypes using two methods for estimating the IGR: ASPECTS decay per hour on NCCT, and core volume growth over time on CTP. Our findings indicate that the NCCT-based definitions of progressor phenotypes were consistently associated with functional outcomes, whereas no significant association was observed with CTP-based definitions.

In our cohort, faster ASPECTS decay per hour was significantly associated with lower odds of favorable functional outcomes and higher risks of mortality and hemorrhagic complications. Specifically, we observed ~ 20% lower odds of achieving a good functional outcome for each stepwise increase in stroke progressor phenotype and ~ 5% lower odds for each additional ASPECTS point decay per hour. This points to the potential validity of ASPECTS decay as a pragmatic and accessible surrogate for infarct progression and prognosis, in contrast with previous studies that did not demonstrate its prognostic utility [16, 23]. Importantly, ASPECTS decay should be interpreted as a prognostic marker of infarct progression rather than as a criterion to exclude patients from EVT. Recent randomized trials have demonstrated the benefit of thrombectomy even in patients with low ASPECTS, supporting an expansion of treatment eligibility beyond traditional imaging thresholds [25, 26]. In this context, CTP remains crucial for identifying patients eligible for reperfusion therapies in the extended time window, particularly for intravenous thrombolysis. Of note, previous studies exploring ASPECTS decay have used data-driven thresholds (e.g., tertiles or quartiles of the study cohort) to define progressor groups. In contrast, we applied predefined cutoffs aligned with those used in volumetric assessment of IGR (5 and 10 mL/h), aiming to test externally validated thresholds rather than internal distribution-based criteria. Nonetheless, several limitations of the ASPECTS-based approach should be considered. First, the scoring is subject to inter-rater variability, especially in early phases where hypodensity may be subtle [27]. Second, the assumption that each ASPECTS point equates to ~ 20 mL of infarcted tissue is an approximation that may not apply uniformly. Third, our registry spans from 2011, a period in which patients with ASPECTS < 6 were often excluded from thrombectomy due to limited supporting evidence, potentially introducing a selection bias toward excluding fast progressors. Moreover, the degree of hypodensity—an important marker of infarct severity [28]—was not assessed.

In our cohort, CTP-estimated IGR showed no significant association with outcomes. Although this finding contrasts with some previous studies [15], it may reflect intrinsic limitations of perfusion imaging. The lack of association between CTP-based IGR and clinical outcomes observed in our study should also be interpreted in light of the known heterogeneity of perfusion-derived core estimation. Indeed, core volume estimation is influenced by several factors, including time from symptom onset, post-processing software, threshold selection, and individual physiological variability. In addition, patients with large estimated core volumes represent a particularly heterogeneous group in real-world practice, as treatment decisions are often individualized across centers [20]. Moreover, although we used relative cerebral blood flow < 30% as a definition of ischemic core, alternative thresholds or parameters may yield different estimates of core volume. Perfusion imaging offers a hemodynamic snapshot that may not correspond with irreversible tissue damage [29]. Furthermore, perfusion-estimated core may underestimate the true infarct extent in metabolically vulnerable patients, where altered factors beyond perfusion compromise tissue ischemic resilience [11]. In addition, CTP-based IGR is intrinsically dependent on onset-to-imaging time. In our cohort, onset-to-imaging time differed substantially across CTP-based progressor groups, being shorter in fast progressors and longer in slow progressors. This may have introduced a bias. Moreover, CTP may be preferentially performed in patients suspected to have slower infarct progression, such as those presenting in extended time windows or with more favorable clinical-imaging profiles. Conversely, patients with clearly large infarct cores on NCCT may not undergo further perfusion imaging. This may result in a relative overrepresentation of slow progressors in the CTP group. Finally, a crucial point is that it remains unclear whether perfusion status on CTP dynamically evolves during infarct progression or remains relatively stable over time [30–32]. The absence of an association between CTP-estimated IGR (core volume divided by time) and outcomes observed in our study supports the latter. In line with this, it has been suggested that perfusion-imaging should be used independently from the time of onset when assessing the IGR [31].

Our findings highlight the heterogeneity in IGR assessment and stroke progressor phenotypes classification across imaging modalities in line with a previous study [23]. Accurately identifying stroke progressor phenotypes may have important clinical implications, such as informing prognosis and adjunctive strategies such as neuroprotection. ASPECTS decay, being rapidly calculable on standard non-contrast CT, provides a low-cost, widely available tool for this purpose—even in resource-limited settings. Its integration into trials design could refine treatment prioritization and target populations likely to benefit from therapies aimed at slowing infarct progression. Automated segmentation tools that quantify baseline NCCT hypodensity volumes and degree are needed to provide more accurate and standardized assessment of the IGR on NCCT imaging.

This study has several limitations. First, the cohort included only EVT-treated patients, introducing selection bias toward more favorable imaging and clinical profiles—i.e., more slow progressors. Second, perfusion imaging was available only in a minority of cases, limiting power and generalizability for perfusion-based analyses. In addition, the use of CTP was not standardized across centers and may have been influenced by local protocols, resource availability, and clinical decision-making. Therefore, patients undergoing CTP may represent a selected subgroup, potentially including more complex cases or those evaluated in centers with advanced imaging capabilities, while NCCT is more broadly applied across different clinical settings. This may have introduced selection bias and should be considered when interpreting comparisons between NCCT-based and CTP-based approaches. However, this variability also reflects real-world clinical practice. In this context, our findings may suggest that NCCT-based markers, being widely available and consistently applied, provide a more robust and generalizable assessment of infarct progression, whereas CTP-based estimates may be more influenced by selection and contextual factors. Third, we assumed linear infarct progression, which likely oversimplifies infarct dynamics that is known to be non-linear in fast progressors [31]. Fourth, although collateral circulation is a known determinant of infarct growth, we were unable to explore its association due to the high rate of missing data. This represents an important limitation, as collateral status may partly explain the observed variability between NCCT-based and CTP-based estimates of infarct progression. Future studies should investigate the relationship between ASPECTS decay and collateral grade, to better understand whether NCCT-based markers may indirectly capture the effect of collateral circulation on infarct dynamics. Moreover, tissue-level collateral status—as captured by the hypoperfusion intensity ratio (HIR), a parameter known to correlate strongly with infarct growth and clinical outcomes [7]—was not assessed in our study. Finally, local image reading introduces variability in ASPECTS scoring and perfusion post-processing, especially due to the absence of a core imaging laboratory.

## Conclusion

In this large, real-world cohort of stroke patients treated with EVT, stroke progressor phenotypes assessed with NCCT (ASPECTS decay per hour) were associated with functional outcomes, whereas CTP-based definitions (CTP-estimated core volume divide by time) were not. These findings highlight differences in how infarct progression is captured by the two imaging approaches and suggest that NCCT-based markers may provide a robust and widely applicable prognostic assessment in real-world settings since the use of imaging techniques that directly visualize cerebral ischemic changes—such as ASPECTS decay—is a reliable biomarker for infarct progression. In contrast, perfusion-based estimates of core volume adjusted for time from onset were not associated with progression in our cohort. This discrepancy may reflect the relatively stable nature of perfusion status despite ongoing infarction, as well as individual variability in ischemic tolerance. Importantly, these findings should not be interpreted as an alternative to CTP in acute stroke management, as perfusion imaging remains essential for treatment selection and clinical decision-making. These findings highlight the need to develop and validate more sensitive perfusion-based markers capable of capturing dynamic infarct evolution over time. Future studies should validate these findings in broader, unselected stroke populations and explore the role of multimodal imaging markers to further refine stroke progressor phenotyping and its clinical implications.

## Supplementary information

Below is the link to the electronic supplementary material.

Supplementary Material 1
