# Supplementary material for: Clinical Implications of Stroke Progressor Phenotypes defined based on ASPECTS decay and perfusion estimated infarct growth rate: insights from a large national thrombectomy registry
Source: Neurol Sci. 2026 Jun 16;47(7):569. doi: 10.1007/s10072-026-09148-4 (PMC13269382; doi:10.1007/s10072-026-09148-4)
Supplement: Supplementary file 1 — Supplementary Material 1 [file 10072_2026_9148_MOESM1_ESM.docx]

**Clinical Implications of Stroke Progressor Phenotypes defined based on ASPECTS Decay and Perfusion estimated Infarct Growth Rate: Insights from a Large National Thrombectomy Registry.**

**Table S1. Perfusion Imaging specification, n (%)**

|  | Centers (n=28) | Patients (n=897) |
| --- | --- | --- |
| Software |  |  |
| RapidAI | 8 (28.6%) | 602 (67.1%) |
| Siemens Syngo.via | 11 (39.3%) | 147 (16.4%) |
| GE ADW | 5 (17.9%) | 43 (4.8%) |
| Olea | 1 (3.6%) | 11 (1.2%) |
| Brainomix | 2 (7.1%) | 92 (10.3%) |
| Viz.ai | 1 (3.6%) | 2 (0.2%) |

**Table S2. Missing Values, n (%)**

|  | ASPECTS decay (n=8076) | IGR (n=897) |
| --- | --- | --- |
| Age | 0 (0.0%) | 0 (0.0%) |
| Sex (female) | 0 (0.0%) | 0 (0.0%) |
| Previous TIA/Stroke | 0 (0.0%) | 0 (0.0%) |
| Atrial Fibrillation | 0 (0.0%) | 0 (0.0%) |
| Diabetes | 0 (0.0%) | 0 (0.0%) |
| Hypertension | 0 (0.0%) | 0 (0.0%) |
| Coronary Artery Disease | 0 (0.0%) | 0 (0.0%) |
| Valvulopathy | 0 (0.0%) | 0 (0.0%) |
| Heart Failure | 0 (0.0%) | 0 (0.0%) |
| Smokers (current or past) | 0 (0.0%) | 0 (0.0%) |
| Hyperlipemia | 0 (0.0%) | 0 (0.0%) |
| Carotid Atherosclerosis | 0 (0.0%) | 0 (0.0%) |
| Neoplasia | 0 (0.0%) | 0 (0.0%) |
| Antiplatelet therapy | 0 (0.0%) | 0 (0.0%) |
| Anticoagulant therapy | 0 (0.0%) | 0 (0.0%) |
| Antihypertensive therapy | 0 (0.0%) | 0 (0.0%) |
| Statins | 0 (0.0%) | 0 (0.0%) |
| Baseline NIHSS | 148 (1.8%) | 5 (0.6%) |
| Intravenous thrombolysis | 247 (3.1%) | 20 (2.2%) |
| TICI 2b-3 | 151 (1.9%) | 7 (0.8%) |
| Onset-to-Imaging | 0 (0.0%) | 0 (0.0%) |
| Onset-to-Recanalization | 429 (5.3%) | 68 (7.6%) |
| ASPECTS | 0 (0.0%) | 248 (27.6%) |
| Core Volume | 7425 (91.9%) | 0 (0.0%) |
| Hypoperfusion Volume | 7430 (92.0%) | 13 (1.4%) |
| IGR | 7425 (91.9%) | 0 (0.0%) |
| Collaterals assessment | 4765 (59%) | 662 (73.8%) |
| mRS | 479 (5.9%) | 23 (2.6%) |
| Any ICeH | 253 (3.1%) | 17 (1.9%) |
| sICeH | 253 (3.1%) | 17 (1.9%) |
